# Endo-MitoEGFP Mice: A Novel Transgenic Mouse with Fluorescently Marked Mitochondria in Microvascular Endothelial Cells

**DOI:** 10.1371/journal.pone.0074603

**Published:** 2013-09-03

**Authors:** Sarah Pickles, Maxime Cadieux-Dion, Jorge I. Alvarez, Marc-Andre Lécuyer, Sarah L. Peyrard, Laurie Destroismaisons, Lydia St-Onge, Simone Terouz, Patrick Cossette, Alexandre Prat, Christine Vande Velde

**Affiliations:** 1 Centre d’Excellence en Neuromique de l’Université de Montréal, Centre de Recherche du Centre Hospitalier de l’Université de Montréal (CRCHUM), Université de Montréal, Montréal, Quebec, Canada; 2 Department of Biochemistry, Université de Montréal, Montréal, Quebec, Canada; 3 Department of Medicine, Université de Montréal, Montréal, Quebec, Canada; Institute of Neurology (Edinger-Institute), Germany

## Abstract

Blood vessel-specific fluorescent transgenic mice are excellent tools to study the development of the vasculature and angiogenic processes. There is growing interest in the biological processes relevant to endothelial cells but limited tools exist to selectively evaluate subcellular functions of this cell type *in vivo*. Here, we report a novel transgenic animal model that expresses mitochondrially targeted enhanced green fluorescent protein (EGFP) via the Hb9 promoter, a homeobox transcription factor with limited known involvement in the vasculature. Random integration of the transgene, containing the entire mouse Hb9 promoter, was found to be expressed in a variety of vascularised tissues. Further inspection revealed that Mito-EGFP localizes to the endothelial cells (ECs) of a subset of microvascular blood vessels, especially in the central nervous system (CNS), heart, spleen, thymus, lymph nodes and skin. We demonstrate the utility of this novel transgenic mouse, named Endo-MitoEGFP, in the detection, imaging, and isolation of microvascular ECs and evaluation of EC mitochondrial function isolated from adult animals. These transgenic mice will be useful to studies of ECs in development, physiology, and pathology.

## Introduction

Transgenic reporter mice are important tools to study biological processes. Fluorescent transgenic mice have been previously developed to study blood vessels (*Tie2*-GFP) [1] and the lymphatic system (*Prox1*-GFP) [2]. A recent description of *Prox1*-GFP mice, a homeobox transcription factor that has widespread CNS expression [3], was also found to have unique expression in the lymphatic system and was excluded from blood vascular endothelial cells [2]. These models allow for studies *in vivo*, as well as the possibility for cell isolation. The current paradigm to achieve cell-specific expression of a reporter protein uses transcription factors, many coming from the homeobox family. Indeed, these transcription factors often serve as cell fate markers, however our understanding of their expression and regulation in development or in adult tissues is not complete. In fact many homeobox proteins with “neuronal restricted” expression have been found to also be expressed in other tissues and cell types [4–6].

In the current report, transgenic mice with a novel vasculature expression pattern were created by random integration of cDNA encoding mitochondrially targeted EGFP under the control of the homeobox transcription factor *Hb9*, a well-established specification factor for motor neurons. Mitochondrial localization of EGFP was achieved via the inclusion of the mitochondrial targeting sequence of a subunit of the electron transport chain fused to EGFP. We provide immunofluorescent, immunoblot and flow cytometric analysis of these mice, establishing unexpectedly, the expression of EGFP within ECs of vessels and have aptly named these mice Endo-MitoEGFP. We propose that the Hb9 promoter has come under altered or previously uncharacterized regulation during random integration of the transgene, leading to a novel expression pattern. We predict that the isolation and evaluation of mitochondrial function from ECs will be greatly aided by the use of the Endo-MitoEGFP transgenic model. Moreover, this model provides a unique opportunity to study the contribution of mitochondria to EC development, normal physiology, and in pathological conditions. Our data demonstrate the experimental usefulness of this novel transgenic model.

## Results and Discussion

**Figure 1:**
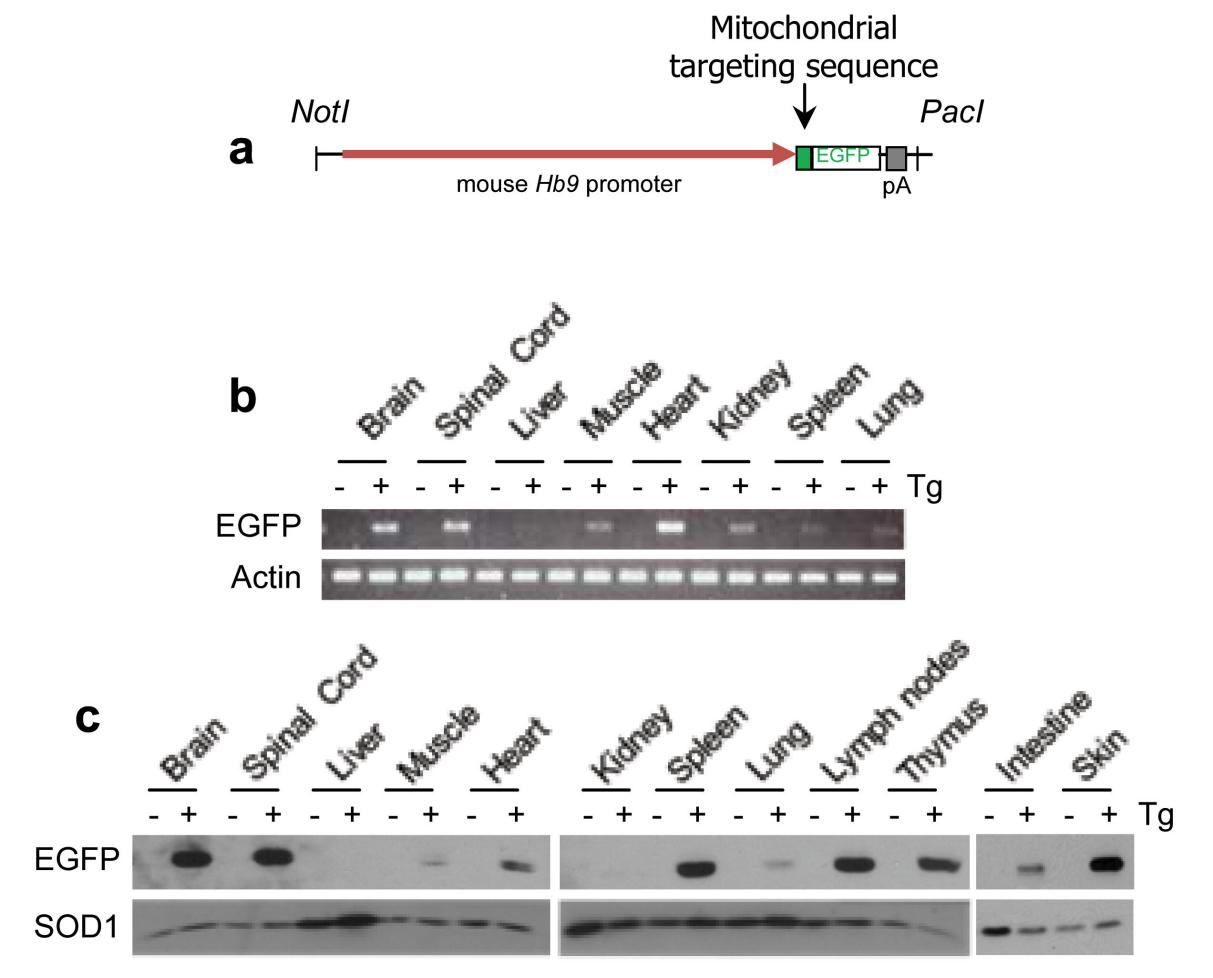
EGFP expression is not restricted to central nervous system, but is also expressed in vascularized tissues. (A) Schematic of p*Hb9*-MitoEGFP transgenic construct, with mitochondrial targeting sequence of Cytochrome *c* Oxidase subunit VIII. (B) RT-PCR of EGFP mRNA from a panel of tissues of transgenic (+) and non-transgenic littermates (-). Actin serves as a loading control. (C) EGFP protein levels detected via immunoblotting in a panel of tissues isolated from transgenic (+) and non-transgenic littermates (-). SOD1 serves as a loading control. *n*=3-4 animals.

A transgene encoding mitochondrially targeted EGFP (MitoEGFP) expressed from the promoter of the mouse homeobox transcription factor Hb9 was introduced via pronuclear injection and randomly integrated into the mouse genome as expected (Figure **1A**). Hb9 is typically expressed in post-mitotic motor neurons of the spinal cord during development, is required for the maintenance of motor neuron identity, and as such, is well regarded as a marker for motor neurons [7,8]. During the initial characterization of founder mice which genotyped positively for the transgene, it was noted that while several founders had the expected motor neuron-restricted expression of EGFP-labelled mitochondria [9], one founder exhibited a unique expression profile which extended beyond the central nervous system (CNS). Specifically, transgene mRNA was detected at high levels in brain, spinal cord, and heart with lesser amounts detectable in gastrocnemius muscle, kidney, spleen and lung and was largely absent in liver (Figure **1B**). Evaluation of EGFP protein levels revealed a similar pattern with the highest levels detected in the brain, spinal cord, spleen, lymph nodes, thymus and skin (Figure **1C**). Modest EGFP expression was detected in muscle, heart, lung, and intestine, but was absent by immunoblot in liver and kidney (Figure **1C**).

**Figure 2:**
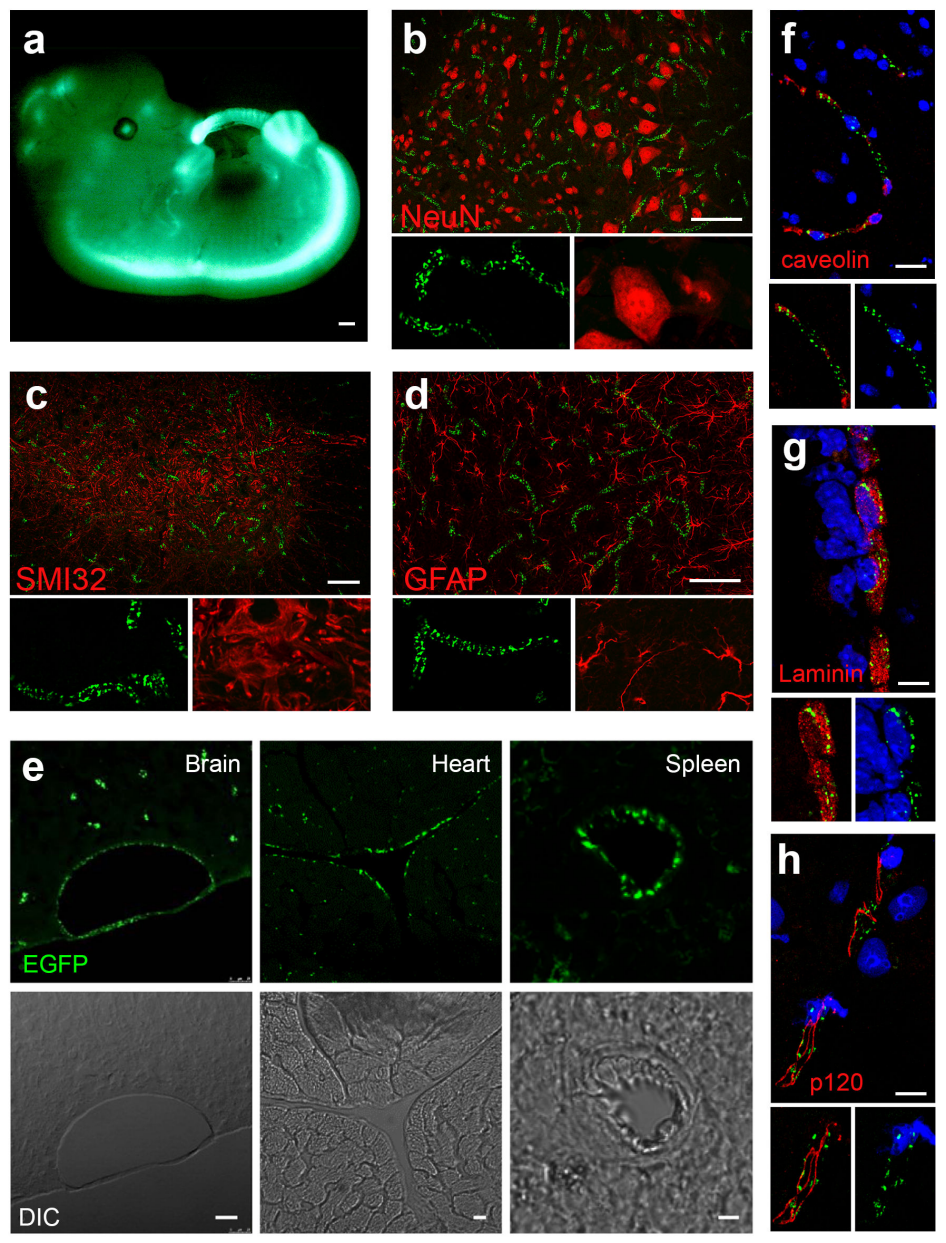
EGFP is expressed in endothelial cells (EC) of blood vessels. (A) Low power magnification of 12.5 day old embryo. Scale bar represents 250 µm. High power magnifications of lumbar spinal cord sections showing expression of EGFP (*green*) does not co-localize with (B) NeuN (*red*), a pan-neuronal marker. (C) unphosphorylated neurofilament, SMI32 (*red*), a motor neuron marker or (D) expression of the astrocyte marker GFAP (*red*). Scale bar represents 75 µm. (E) Higher power magnifications of brain (*left*), heart (*middle*) and spleen (*right*) sections, showing EGFP expression (*green*) and transmitted light (*DIC*). Scale bar represents 25, 10 and 5 µm for the brain, heart, and spleen, respectively. Brain sections showing expression of EGFP (*green*), and (F) EC marker caveolin (red), (G) matrix marker laminin (*red*) and (H) junctional protein p120 (*red*). Nuclei are marked by TOPRO-3 (blue). Scale bar represents 15 µm (F and G) and 105 µm (H). *n*=2 animals.

To determine the cell type in which EGFP protein was expressed, we examined native EGFP expression in a panel of tissues via confocal microscopy. During embryogenesis, EGFP expression was detected in the motor cortical strip and spinal cord, as expected for Hb9 (Figure **2A**). However, in adult mouse spinal cord EGFP expression was absent from motor neurons and other spinal neurons, as evidenced by the lack of co-localization of EGFP with NeuN or unphosphorylated neurofilament (SMI32), pan-neuronal and motor neuron markers, respectively (Figure **2B, 2C**). EGFP expression was also absent from astrocytes as marked with the astrocytic marker GFAP (Figure **2D**). During this initial analysis, we noted that EGFP was expressed in a speckled pattern within filamentous looking structures resembling blood vessels. Given the high level of EGFP expression in the brain, heart and spleen we examined these tissues and determined that this pattern was reminiscent of the vascular endothelium (Figure **2E**). Co-labelling with caveolin, the vessel matrix protein laminin and EC marker p120-catenin [10,11], demonstrated EGFP localization to the vasculature/ECs in the brain (Figure **2F–H**). EGFP was primarily localized to small parenchymal vessels, mainly capillaries, with only modest labelling of larger vessels and was undetectable in the ECs lining the arteries and larger meningeal vessels.

**Figure 3:**
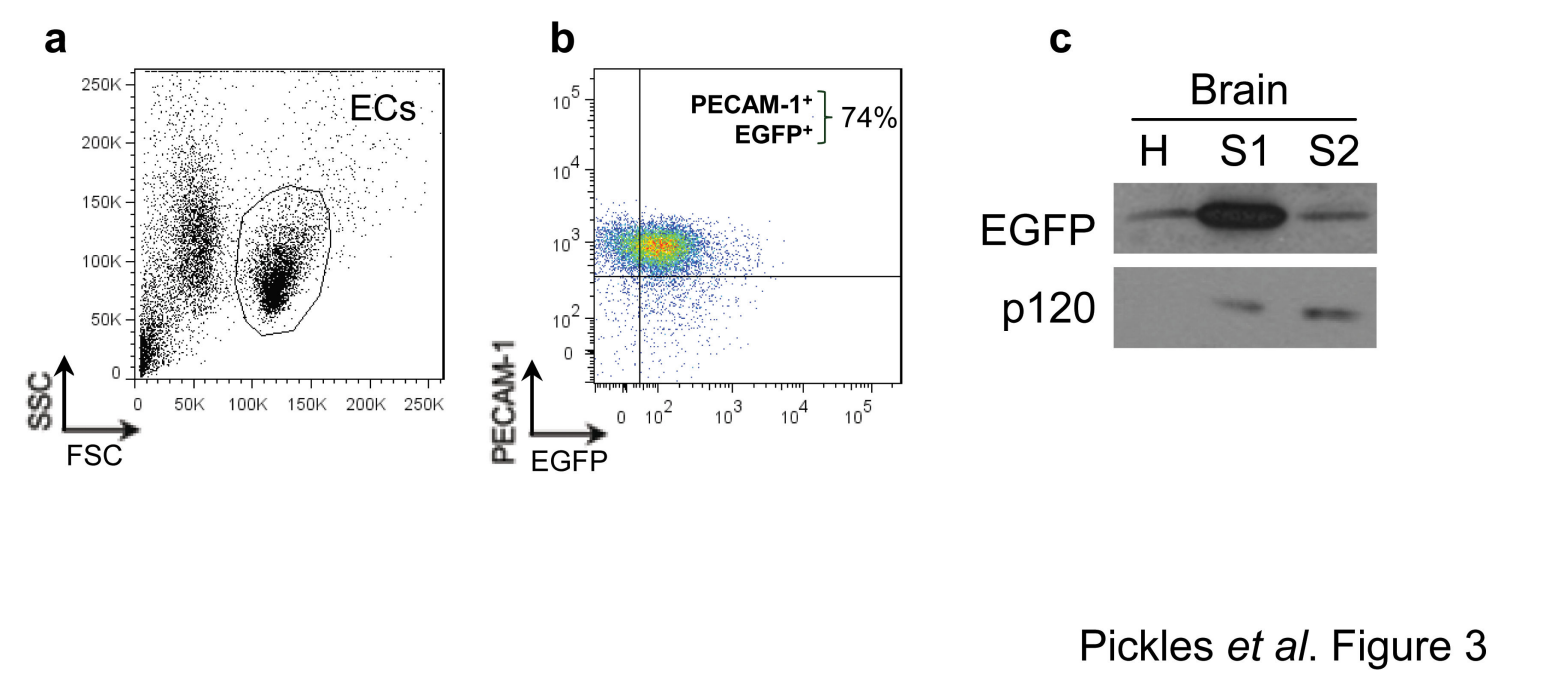
EGFP expression is detected in ECs via flow cytometry. (A) Representative dot plot of ECs isolated from spleens of transgenic mice. The EC population was selected based on size and labelling with PECAM-1. (B) The majority of splenic ECs express EGFP and PECAM-1. (C) Western blot of brains homogenates (H), as well as soluble (S1) and insoluble (S2) fractions from lysates of ECs isolated from CNS vessels of transgenic animals and immunoblotted for EGFP and p120. *n*=3 animals.

To further prove that EGFP expression was restricted to ECs, splenic ECs were isolated from transgenic animals and labelled with fluorescently conjugated PECAM-1 antibody and analyzed by flow cytometry. PECAM-1 is expressed at the surface of ECs and is a well-recognized EC marker [12]. Two distinct cell populations were identified based on light scattering properties and ECs were identified by their larger size and positive cell surface labelling for PECAM-1 (Figure **3A**). Within the splenic EC population, 81.3 ± 5.8% stained positive for PECAM-1 and 74.0 ± 10.5% of these cells also expressed EGFP (Figure **3B**), indicating that a majority of ECs within the spleen express EGFP. While the analysis of brain ECs by flow cytometry was not possible due to low EC yields from brain, we did observe that EGFP was enriched in brain EC fractions as demonstrated by immunoblotting for the junctional protein p120 [13] (Figure **3C**).

**Figure 4:**
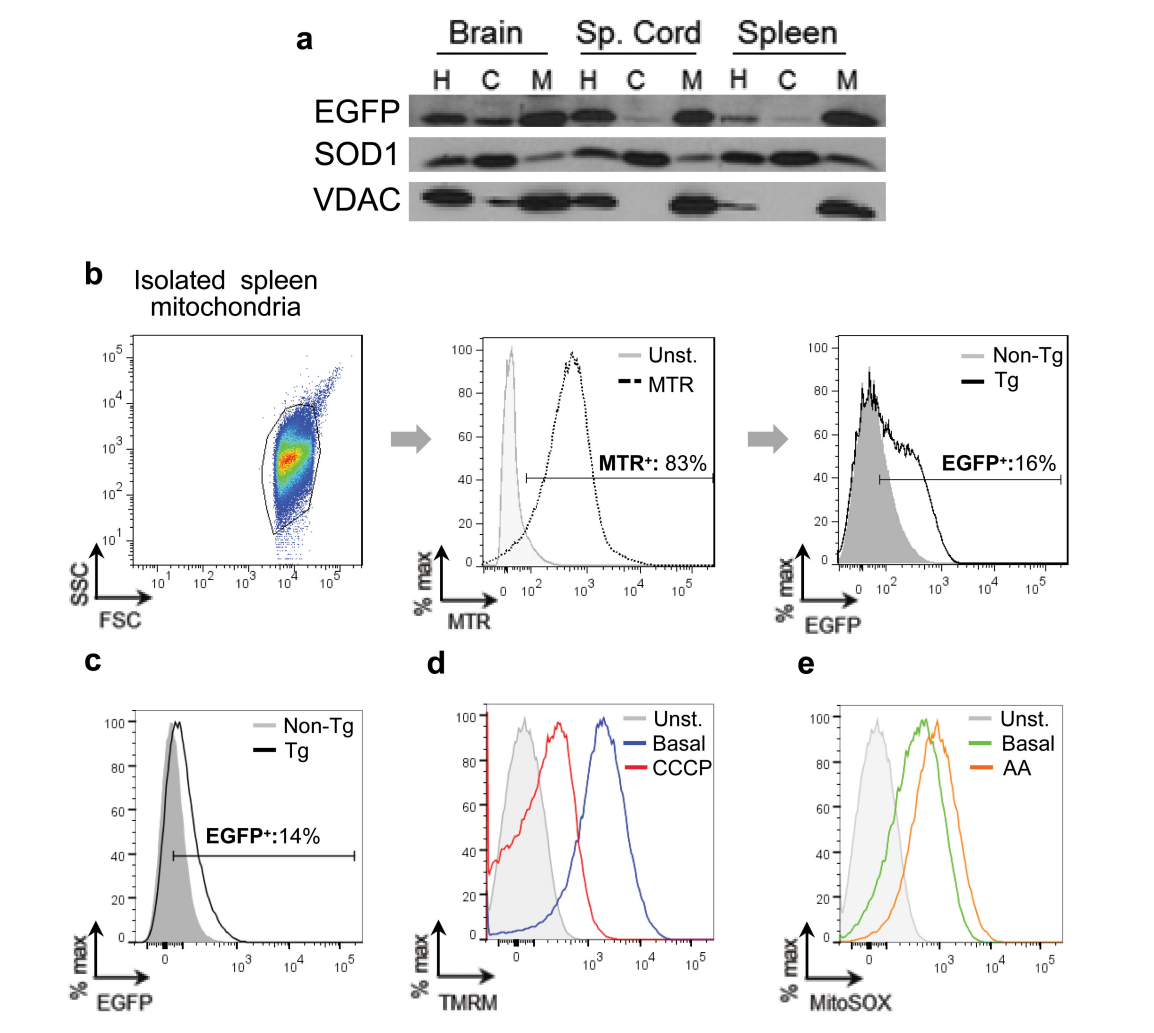
EGFP is expressed within mitochondria. (A) Homogenates (H), cytosolic protein (C) and purified mitochondria (M) were collected from the brain, spinal cord and spleen of transgenic mice and probed for EGFP via immunoblot. SOD1 and VDAC serve as controls for cytosol and mitochondria, respectively. (B) Mitochondria were isolated from the spleens of transgenic and non-transgenic mice. *Left*: Mitochondria were gated according to their light scattering properties (forward scatter, FSC; side scatter, SSC). *Middle*: Gated mitochondria were stained with MitoTracker Red (MTR, black, dashed) and compared to unstained (grey, shaded) mitochondria. *Right*: MTR^+^ mitochondria from transgenic (black, unshaded) and non-transgenic (grey, shaded) mice were analyzed for EGFP expression. Data presented is representative of three independent experiments. (C) Mitochondria from transgenic (black, unshaded), and non-transgenic (grey, shaded) mice were analyzed for EGFP expression. (D) The transmembrane potential of EGFP^+^ mitochondria was assayed using TMRM. Mitochondria were left, unstained (grey, filled), treated with TMRM, basal conditions (blue), or treated with the protonophore CCCP (red). (E) Mitochondrial superoxide production of EGFP^+^ mitochondria, was assayed using MitoSOX Red. Mitochondria were left, unstained (grey, filled), treated with MitoSOX Red under basal conditions (green), or treated with the complex III inhibitor Antimycin A (orange). C, D, E are from the same sample and are representative of three independent experiments.

In order to verify that EGFP protein was targeted to mitochondria as expected, mitochondria were isolated from brain, spinal cord and spleen via differential centrifugation. As expected, EGFP was predominately localized to fractions enriched for mitochondria in all tissues examined (Figure **4A**). SOD1 and VDAC serve as markers for the cytosolic and mitochondrial fractions, respectively. EGFP expression in spleen mitochondria was also examined by flow cytometry. Spleen homogenates, containing every cell in the spleen including endothelial cells, were processed for mitochondrial isolation. Mitochondria were initially selected based on size, as reflected by forward and side light scatter (FSC, SSC), and positive labelling with the mitochondria specific dye MitoTracker Red (MTR) (Figure **4B**). An average of 83.5 ± 4.7% of the total events collected were MTR^+^, and thus mitochondria. Of these, 16.5 ± 4.1% of these events exhibited EGFP expression (Figure **4B**). To demonstrate the utility of this model for the evaluation of endothelial mitochondrial function, isolated spleen mitochondria were labelled with fluorescent indicator dyes reporting on different aspects of mitochondrial function by flow cytometry, and mitochondria that expressed EGFP were selected for evaluation (Figure **4C**). [In these experiments, mitochondrial identity was confirmed in a separate sample using MitoTracker Green, a dye that selectively accumulates in mitochondria (data not shown)]. The separation of charge across the mitochondrial inner membrane, referred to as the mitochondrial transmembrane potential (ΔΨ_m_) is generated by the pumping of hydrogen atoms out of the matrix by members of the electron transport chain. This proton gradient is essential for the production of ATP, and thus serves as an excellent way to evaluate mitochondrial function. The fluorescent dye Tetramethylrhodamine, methyl ester (TMRM) is selectively taken up by mitochondria in proportion to the mitochondrial transmembrane potential. In our experiments, nearly all EGFP^+^ spleen mitochondria are TMRM^+^ (89.6 ± 0.8%), as expected for healthy mitochondria (Figure **4D**). EGFP^+^ mitochondria also responded characteristically to Carbonyl cyanide *m*-chlorophenyl hydrazone (CCCP), a protonophore that allows the hydrogen ions to pass freely across the inner mitochondrial membrane, thereby collapsing the electrochemical gradient, causing depolarization and a corresponding decrease in TMRM fluorescence. Experimentally, this is reflected in a decreased percentage of mitochondria (43.0 ± 4.0%) falling within the gate previously determined by TMRM staining (Figure **4D**). The mitochondrial transmembrane potential of EGFP^+^ mitochondria was not significantly different from EGFP^-^ mitochondria within the same sample or from non-transgenic littermate controls. Similarly, the response to CCCP was unaltered by the presence of EGFP (data not shown).

Mitochondria normally produce superoxide as a by-product of oxidative phosphorylation. Mitochondrial superoxide can be specifically measured with MitoSOX Red, which produces a red fluorescent signal when oxidized. As expected, EGFP^+^ mitochondria produce superoxide (66.5 ± 2.7% fall within the predetermined gate). An increased proportion of mitochondria label positively with MitoSox Red when mitochondria are treated with the complex III inhibitor antimycin A (AA; 75.2 ± 1.4%) (Figure **4E**). As was observed above, a comparison of superoxide production of EGFP^+^ mitochondria and EGFP^-^ mitochondria from non-transgenic littermate controls revealed no statistically significant difference (data not shown). Taken together, these mice represent a novel tool with which to evaluate key functional features of endothelial mitochondria isolated from vascularised tissues.

Hb9 is a well described homeobox transcription factor best characterized for its role in motor neuron development and in axonal pathfinding for a subset of neurons [7,14,15]. This promoter has been extensively used to generate motor neuron restricted EGFP expression in other transgenic models including mice, fly, zebrafish and chick [7,14]. During the characterization of a similarly intended transgenic line [9], we serendipitously generated the Endo-MitoEGFP model, where expression of EGFP within mitochondria was absent from the intended cell type but presented with a novel microvascular pattern consistent with expression within a subset of endothelial cells. Although Hb9 is often considered exclusively as a marker of motor neurons, it is well published that Hb9 is widely expressed in the endoderm during development which gives rise to the respiratory and digestive tubes. Furthermore, Hb9 is essential for early differentiation of the dorsal gut epithelium into pancreatic tissue and is also detected in differentiated beta cells [16,17]. An early characterization of the Hb9 transcript in human tissues reported expression in colon, small intestine, pancreas, lymphoid tissues and a range of hematopoetic cell lines [18]. Interestingly, Hb9 expression is also well reported in human bone marrow, especially in CD34^+^ cells, and it becomes downregulated following differentiation [18,19]. Therefore, expression of Hb9 is not solely restricted to motor neurons and is more broadly expressed in other tissues during development and adulthood.

The mechanism(s) which regulate Hb9 expression are not fully understood, especially in non-motor neuron cell types [20]. Interestingly, Hb9 expression was increased 12.5 fold in primary human endothelial cells derived from umbilical vein following treatment with the pro-angiogenic sulfated steroid, sokotrasterol, suggesting a novel role for Hb9 in endothelial cell sprouting and/or angiogenesis [21]. In addition, it is noteworthy that the development of the vascular and neuronal systems are closely coupled in their genesis and branching/path formation [22–24]. In fact, the vascular and nervous systems are the first tissue systems specified during development [24]. The coordinated and concomitant expression of a variety of homeobox factors is required for angiogenesis in the CNS [22]. Homeobox factors are also required for formation of the cardiovascular and lymphatic system. For example, several genes in the HOX gene cluster including HOXA5, HOXA11, HOXB1, HOXB7, and HOXC9, are expressed during the development of the cardiac system; and Prx1 is involved in both cardiac and lymphatic tissue development [4]. In fact, arteries follow the path of nerves in embryonic limb skin, due to expression of VEGF in peripheral nerves and Schwann cells which induces arterial marker expression in endothelial cells [25]. Ablation of peripheral nerves due to genetic deletion of Neurogenin 1 and 2 yields defects in artery branching [25]. Isl1, another homeobox protein is also involved in the specification and maintenance of neuronal identities (especially motor neurons) [26], is now also well known to be expressed during development in the second heart field, giving rise to key structures within the heart [5,6]. In addition, embryonic stem cells expressing Isl1 *in vitro* can differentiate into cardiac progenitors as well as endothelial cells [27], leading to speculation that the expression of this transcription factor may be more widespread than previously believed [28]. Indeed, a careful characterization of Isl1 reporter mice led to the discovery that Isl1 cells are present in the endothelium of the aorta and in umbilical vessels [28]. Taken together, these studies demonstrate the connection between homeobox transcription factors, the development of the vasculature and endothelial cells. We propose here that random integration of the Hb9 promoter in the Endo-MitoEGFP mouse has come under novel or previously uncharacterized regulation leading to expression in a subset of ECs within a variety of vascularized tissues. We speculate that like Isl1 and other homeobox transcription factors, Hb9 may play a dual role in motor neuron identity and development of the vascular system.

## Conclusions

In summary, we report the development of Endo-MitoEGFP mice which feature mitochondrial-restricted expression of EGFP in microvascular ECs. These mice will be instrumental in examining the role and function of mitochondria in EC development, normal adult physiology, and potentially in certain pathologies such as arthrosclerosis, diabetes, multiple sclerosis, Alzheimer’s disease, and amyotrophic lateral sclerosis.

## Materials and Methods

### Generation of transgenic mice

Animals used in this study were treated in strict accordance to a protocol (N08001CVsr) approved by the Centre de Recherche du Centre Hospitalier de l’Universite de Montreal (CRCHUM) Institutional Committee for the Protection of Animals which follows national standards as outlined by the Canadian Council on Animal Care (CCAC). The transgenic vector p*Hb9*-MitoEGFP was generated by introducing the mitochondrial targeting sequence of Cytochrome *c* Oxidase subunit VIII into p*Hb9*-EGFP (Dr. Sam Pfaff, Salk Institute) via standard cloning techniques. Transgenic founders (from F1 C57Bl/6 parental mice) were generated by pronuclear injection of a 10 kb NotI*-*PacI fragment containing p*Hb9*-MitoEGFP. Five founders were identified via PCR, one of which gave a staining pattern that resembled the vasculature. These mice, referred to as Endo-MitoEGFP, were backcrossed with C57Bl/6 for eight generations with each generation yielding a consistent expression pattern. Genotyping was done via PCR, using the following primers: 5’-TCTTCTTCAAGGACGACGGCAACT-3’ and 5’-CCTTGATGCCGTTCTTCTGCTTGT-3’, as previously described [9]. Mice with the expected motor neuron-restricted transgene expression have been published elsewhere [9]. Animals were treated in accordance with Canadian Council for Animal Care (CCAC) and the Centre de recherche du Centre hospitalier de l’Université de Montréal (CRCHUM) guidelines.

### RT-PCR

RNA was extracted using the RNAeasy kit (Qiagen) and reverse transcribed with QuantiTect (Qiagen). Resulting cDNA was processed for standard PCR using the following primer sets: EGFP 5C: 5’-TCTTCTTCAAGGACGACGGCAACT-3’; EGFP 3C: 5’-CCTTGATGCCGTTCTTCTGCTTGT-3’; β-actin exon 5 F: 5’-CGTTGGCATCCACGAAACTA-3’; β-actin exon 6 R: 5’-AGTACTTGCGCTCAGGAGGA-3’.

### Immunoblotting

Adult mice were euthanized with isofluorane and then transcardially perfused with cold PBS prior to tissue collection. Tissues were homogenized in 5 volumes of lysis buffer (50 mM Tris pH 7.5, 1 mM EDTA, 150 mM NaCl, 1% NP-40, 1% SDS, and protease inhibitors). Cleared tissue lysates (25µg), isolated brain ECs (10µg) or mitochondrial and cytosolic fractions were subjected to SDS-PAGE and immunoblotted with an in-house polyclonal anti-EGFP antibody generated against the full-length protein (Covance), SOD1 (Stressgen), VDAC (Calbiochem) and p120 (BD Biosciences).

### Immunofluorescence

Animals were transcardially perfused with 4% phosphate-buffered paraformaldehyde (FD NeuroTechnologies). Tissues were subsequently dissected, post-fixed for 2 hours, cryoprotected, and then embedded in OCT (TissueTek). Ten micron sections collected directly on slides were blocked with 3% BSA in 0.2% Triton X-100/PBS, labelled with, SMI32 (Covance), NeuN (Millipore), GFAP (DAKO) diluted in blocking buffer. Antibodies were visualized via fluorescently conjugated anti-mouse or anti-rabbit secondary antibodies (Texas Red; Jackson Immunochemicals). Stainings for caveolin (Santa Cruz), laminin (DakoCyomation), and p120 (Santa Cruz) were done on sections from PBS-perfused mice. Slides were fixed with cold ethanol prior to immunolabelling. Coverslips were sealed using ProLong Antifade reagent (Invitrogen) and analyzed with a confocal microscope (Leica SP5; 63x oil, 1.7 NA) and processed with Leica LAS AF software and/or Photoshop CS4 (Adobe).

### Isolation of splenic and brain ECs

Transgenic animals were processed as previously described to isolate spleen and CNS vessels [13]. Briefly, tissues were dissected, minced and homogenized. Homogenates were washed with Hanks Balanced Salt Solution (HBSS) and centrifuged in 30% dextran (Sigma) at 4 000 x *g* for 30 min. For brains, the myelin layer was discarded and the pellet containing the ECs was washed and processed for western blot. Cells were lysed in 10 mM HEPES, 100 mM NaCl, 2 mM EDTA, 1% Triton X-100, pH 7.4 with protease inhibitors, using a 21G needle. Soluble proteins (S1) were obtained by collecting the supernatant after centrifugation at 15 000 x *g*. Insoluble fractions (S2) were resuspended in buffer with 1% SDS, sonicated, and then centrifuged at 15 000 x *g*. For spleens the pellet which contains vascular components was washed in HBSS and processed for flow cytometry.

### Isolation of mitochondria

For western blotting, mitochondria were isolated exactly as previously described [29]. For flow cytometry, mitochondria were isolated from spleen homogenates via differential centrifugation (17 000 x *g*) in homogenizing buffer (HB: 210 mM Mannitol, 70 mM Sucrose, 10 mM Tris, 1 mM EDTA, pH 7.5).

### Flow cytometry

Splenic ECs were labelled for expression of PECAM-1 at the cell surface with monoclonal PECAM-1 APC (BD Bioscience) or isotype control in FACS Buffer (1% Fetal Bovine Serum, 0.1% sodium azide in PBS). EC’s were first gated according to size by light scattering properties (FSC/SSC), then PECAM-1 and EGFP expression were examined. Mitochondria (25 µg) were labelled with MitoTrackerRed (MTR, 100 nM; Invitrogen) to confirm mitochondrial identity in HB Buffer. Native EGFP fluorescence was detected without antibody. For mitochondrial functional assays, mitochondria (25 µg) were incubated in M Buffer (220 mM sucrose, 68 mM mannitol, 10 mM KCl, 5 mM KH_2_PO_4_, 2 mM MgCl_2_, 500 µM EGTA, 5 mM succinate, 2 µM rotenone, 10 mM HEPES pH 7.2, 0.1% fatty acid-free BSA). Tetramethylrhodamine Methyl Ester (TMRM, 100 nM; Invitrogen) was used to assess mitochondrial transmembrane potential (ΔΨ_m_) and MitoSOX Red (MitoSOX, 5 µM; Invitrogen) to quantify mitochondrial superoxide levels. The protonophore carbonyl cyanide *m*-chlorophenyl hydrazone (CCCP, 100 µM; Sigma) was used as a control for ΔΨ_m_ measurements, and the complex III inhibitor, Antimycin A (AA, 100 µM; Sigma) was used as a control for mitochondrial superoxide production. Mitochondria were gated according to light scatter, after doublets were excluded, then EGFP^+^ events were selected, and levels of TMRM and MitoSOX Red were evaluated. Dyes and antibodies selected exhibited distinct spectral properties with minimal to no overlap. Where necessary, compensation was applied according to single-color control samples. ECs and mitochondrial samples were processed on a LSR II flow cytometer (BD Biosciences). All flow cytometry data was analyzed with FlowJo (Treestar, Ashland, OR).
